# Decreased expression of protocadherin 20 is associated with poor prognosis in hepatocellular carcinoma

**DOI:** 10.18632/oncotarget.13822

**Published:** 2016-12-07

**Authors:** Yanqin Wu, Shuhui Zheng, Jiayan Yao, Minrui Li, Guang Yang, Ning Zhang, Shenghong Zhang, Bihui Zhong

**Affiliations:** ^1^ Department of Gastroenterology, The First Affiliated Hospital, Sun Yat-sen University, Guangzhou, 510080, P.R. China; ^2^ Research Center of Translational Medicine, The First Affiliated Hospital, Sun Yat-sen University, Guangzhou, 510080, P.R. China

**Keywords:** protocadherin 20, cadherin family proteins, hepatocellular carcinoma, biomarker, overall survival

## Abstract

Recently, protocadherin 20 has been reported as a tumor suppressor gene in hepatocellular carcinoma (HCC); however, the prognostic value of protocadherin 20 in HCC remains unclear. Hence, the purpose of this study was to investigate the clinical and prognostic values of protocadherin 20 in HCC patients. The expression of protocadherin 20 was assessed by quantitative real-time polymerase chain reaction, western blot, and immunohistochemistry. Kaplan-Meier curves were created to calculate the overall survival of the patients, and Cox regression models were used to identify the risk factors associated with prognosis. Of 317 primary HCC patients, decreased expression of protocadherin 20 was observed in 184 (58.0%) patients (*P <* 0.001). Reduced protocadherin 20 protein expression correlated with portal hypertension, poor tumor differentiation, advanced Okuda stage, and Cancer of the Liver Italian Program score (all *P <* 0.05). Low protocadherin 20 expression was an independent risk factor for mortality (*P* = 0.018). Furthermore, in our newly developed simple risk score based on protocadherin 20, patients with total score > 1.11 showed significantly poorer outcome; and the predictive value of the score was better than the Barcelona Clinic Liver Cancer stage, Okuda stage, and Child-Pugh classification (Harrell's concordance index = 0.614). Taken together, these findings suggest that protocadherin 20 may represent a novel prognostic biomarker for HCC patients.

## INTRODUCTION

Hepatocellular carcinoma (HCC) is one of the most common malignant tumors worldwide. An estimated 782,500 new liver cancer cases and 745,500 liver cancer-related deaths occurred globally in 2012, with approximately 50% of the total number of cases and deaths occurring in China [1]. Unfortunately, most HCC patients are diagnosed at the advanced stages of tumor progression. Moreover, patients of the same stage may exhibit different prognoses [2], owning to differences in various clinicopathological parameters and biomarkers, many of which are still being discovered. As the current biomarkers for HCC remain unsatisfactory, it is imperative to identify novel biomarkers and predictors for this disease.

The protocadherin (PCDH) family, a subfamily of the cadherin family, can be divided into two groups based on the genomic structure: clustered and non-clustered [3]. Recently, several non-clustered PCDHs on chromosome 13q21 (PCDH8 [4–6], PCDH9 [7–9], PCDH10 [10–13], PCDH17 [14–17], and PCDH20 [18–20]) have been reported as candidate tumor suppressor genes in human carcinogenesis. PCDH20 (also known as PCDH13) is a novel protocadherin located at 13q21.2. It comprises six extracellular domains, a single transmembrane region, and distinct cytoplasmic portions [21]. The latest studies showed reduced expressions of PCDH20 in non-small cell lung cancer [18], nasopharyngeal carcinoma [19], and HCC [20].

However, no study has fully evaluated the prognostic role of PCDH20 expression or the association of its protein expression with clinicopathological characteristics in HCC. Hence, the current study aimed to investigate the expression of PCDH20 and its clinical significance in HCC.

## RESULTS

### Decreased PCDH20 mRNA and protein expression in HCC

**Figure 1:**
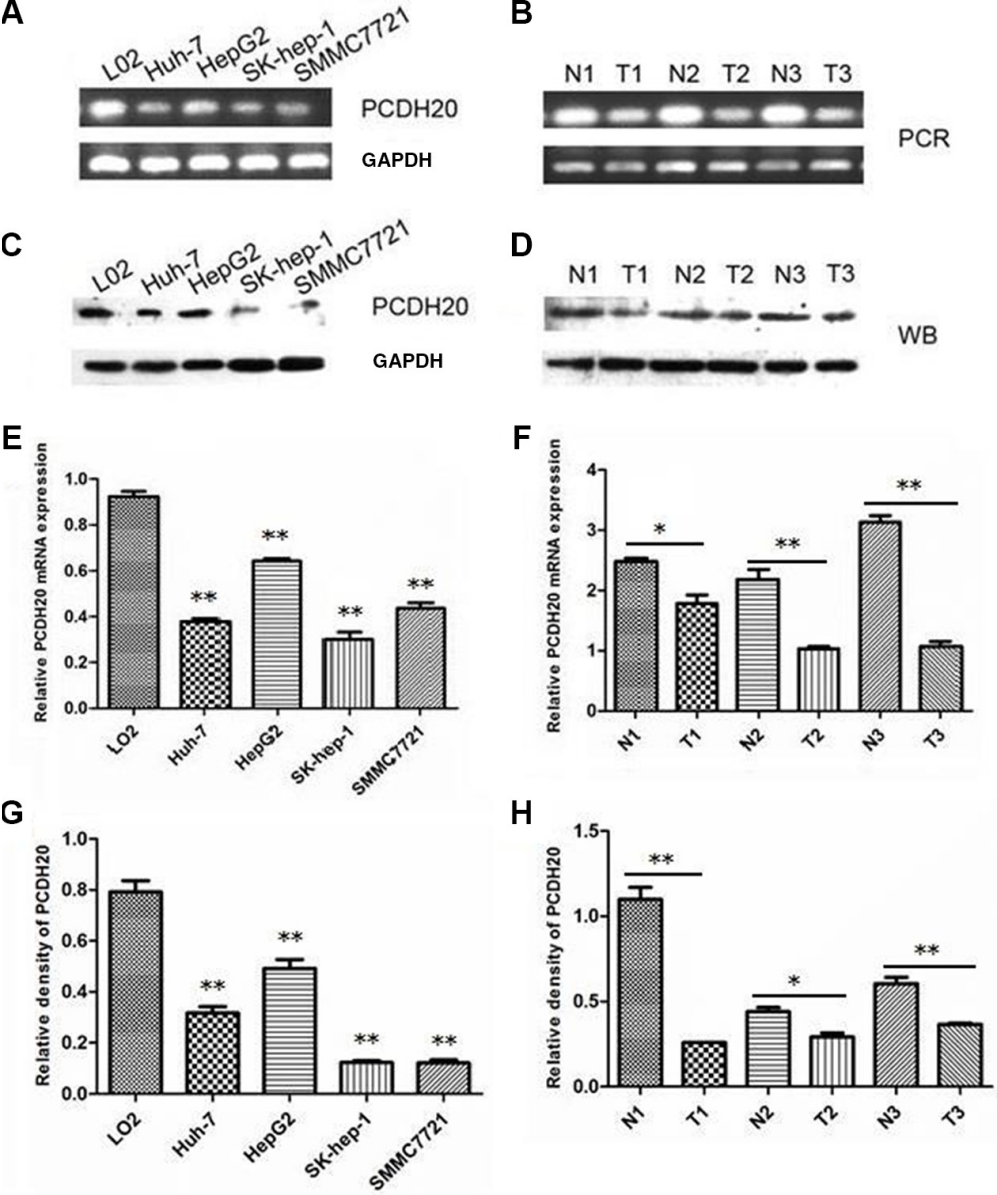
PCDH20 expression in hepatocellular carcinoma cell lines and tissues. (**A**) The mRNA levels of protocadherin 20 (PCDH20) were examined in four human hepatocellular carcinoma (HCC) cell lines and one normal human hepatocyte line by reverse transcription polymerase chain reaction. (**B**) The mRNA levels of PCDH20 were examined in HCC (T) and paracarcinomatous (PC) tissues (N) by reverse transcription polymerase chain reaction. (**C**) Western blot was used to examine the protein levels of PCDH20 in four human HCC cell lines and one normal human hepatocyte line. (**D**) Western blot was used to examine the protein levels of PCDH20 in HCC (T) and PC tissues (N). (**E**) Relative -ΔCt values of the mRNA levels of PCDH20 in four human HCC cell lines and one normal human hepatocyte line by quantitative real-time polymerase chain reaction. (**F**) Relative -ΔCt values of the mRNA levels of HCC and PC tissues by quantitative real-time polymerase chain reaction. (**G**) Relative intensity of the protein levels of PCDH20 in four human HCC cell lines and one normal human hepatocyte line. (**H**) Relative intensity of the protein levels of PCDH20 in HCC and PC tissues. *P < 0.05, **P < 0.01, *t* test.

**Figure 2:**
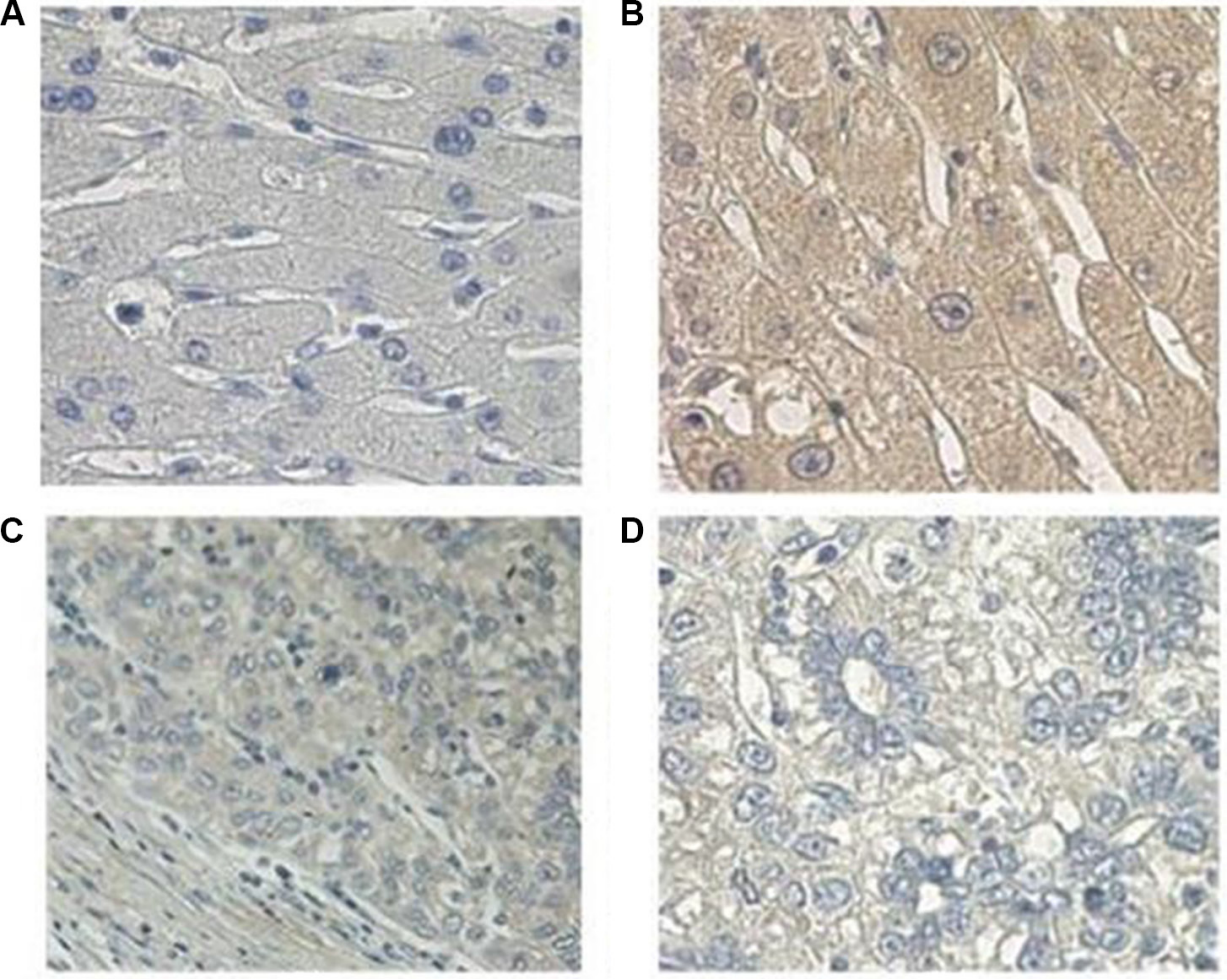
Immunohistochemical analysis of PCDH20 expression in hepatocellular carcinoma. (**A**) Negative control (phosphate-buffered saline instead of anti- protocadherin 20 [PCDH20] antibody, 400×). (**B**) Positive PCDH20 immunohistochemical [IHC] staining of normal liver tissues (400×). (**C**) PCDH20 IHC staining of tumor tissues (200×). (**D**) PCDH20 IHC staining of tumor tissues (400×).

Compared to the immortalized human fetal liver cell line LO_2_, PCDH20 was decreased in four HCC cell lines at both mRNA and protein levels (Figure 1). Immunohistochemistry (IHC) revealed that the PCDH20 expression in the HCC tissues was lower than that in the paracarcinomatous (PC) tissues in 184 of 317 (58.0%) patients (Figure 2). The expression levels of PCDH20 were also tested by quantitative real-time polymerase chain reaction and western blot in 50 patients; and we found that the mean level of PCDH20 expression in the tumor tissues was significantly lower than that in the PC tissues (Figure 1).

### Association between PCDH20 expression and clinicopathological features of HCC patients

The association between PCDH20 expression and clinicopathologic parameters was assessed by chi-square test for proportion, as shown in Tables 1, 2. Low PCDH20 expression was found to correlate with portal hypertension (*P* = 0.041), poor tumor differentiation (*P* = 0.016), advanced Okuda stage (*P* = 0.003), and Cancer of the Liver Italian Program (CLIP) score (*P* < 0.001). No associations were observed between PCDH20 expression and other clinicopathological characteristics.

**Table 1:** Association between PCDH20 expression and clinical parameters

	*n* (%)	PCDH20 expression		*P* value
		Low level (*n=* 184)	High level (*n=* 133)	
Sex				
Male	282 (89.0)	165	117	
Female	35 (11.0)	19	16	0.633
Age (years)				
> 50	181 (57.1)	99	82	
≤ 50	136 (42.9)	85	51	0.163
HBsAg				
Present	262 (82.6)	148	114	
Absent	55 (17.4)	36	19	0.221
Ascites				
Present	62 (19.6)	39	23	
Absent	255 (80. 4)	145	110	0.387
Liver cirrhosis				
Absent to mild	208(65.6)	118	90	
Moderate to severe	109(34.4)	66	43	0.513
Portal hypertension				
Present	20 (6.3)	16	4	
Absent	296 (93.7)	168	128	0.041*
ALT (U/L)				
> 40	156 (49.2)	94	62	
≤ 40	161 (50.8)	90	71	0.432
AST (U/L)				
> 37	195 (61.5)	121	74	
≤ 37	122 (38.5)	63	59	0.068
ALP (U/L)				
> 110	111 (35.0)	67	44	
≤ 110	206 (65.0)	117	89	0.540
GGT (U/L)				
> 50	223 (70.3)	132	91	
≤ 50	94 (29.7)	52	42	0.523
Serum AFP (μg/L)				
> 20	226 (71.3)	130	96	
≤ 20	91 (28.7)	54	37	0.767
Serum CEA (μg/L)				
> 5	35 (13.2)	23	12	
≤ 5	231 (86.8)	133	98	0.362
Serum CA19-9 (U/mL)				
> 35	69 (26.0)	41	28	
≤ 35	196 (74.0)	114	82	0.855
Serum CA125 (U/mL)				
> 35	54 (20.5)	36	18	
≤ 35	210 (79.5)	118	92	0.164

Abbreviations: PCDH20: protocadherin 20; HBsAg, hepatitis B surface antigen; ALT: alanine aminotransferase; AST: aspartate aminotransferase; ALP: alkaline phosphatase; GGT: γ-glutamyltransferase; AFP: alpha-fetoprotein; CEA: carcinoembryonic antigen; CA19-9: carbohydrate antigen 19-9; CA125: carbohydrate antigen 125.

**Table 2:** Association between PCDH20 expression and pathological features and clinical stages

	*n* (%)	PCDH20 expression		*P* value
		Low level (*n=* 184)	High level (*n=* 133)	
Tumor differentiation				
Well to moderately	238 (79.1)	130	108	
Poorly differentiated	63 (20.9)	45	18	0.016*
Tumor number				
Solitary	219 (69.1)	123	96	
Multiple	98 (30.9)	61	37	0.311
Maximum tumor size (cm)				
> 5	277 (88.2)	164	113	
≤ 5	37 (11.8)	17	20	0.125
vascular invasion				
Present	78 (24.6)	47	31	
Absent	239 (75.4)	137	102	0.648
Lymph node metastasis				
Present	44 (14.1)	24	20	
Absent	269 (85.9)	158	111	0.601
**Clinical stages**				
Child classification				
A	266 (83.9)	160	106	
B	47 (14.8)	22	25	0.220
C	4 (1.3)	2	2	
BCLC stage				
0	5 (1.6)	1	4	
A	47 (14.8)	23	24	
B	28 (8.8)	15	13	
C	237 (74.8)	145	92	0.124
Okuda stage				
I	172 (54.3)	87	85	
II	14 (45.7)	97	48	0.003*
III	0			
CLIP score				
0	47 (14.8)	18	29	
1	95 (30.0)	42	53	< 0.001*
2	71 (22.4)	49	22	
3	69 (21.8)	48	21	
4	31 (9.8)	24	7	
5	4 (1.3)	3	1	
6	0			
TNM stage				
I-II	143 (45.1)	77	66	
III-IV	174 (54.9)	107	67	0.170

Abbreviations: PCDH20: protocadherin 20; BCLC: Barcelona Clinic Liver Cancer (staging system); CLIP: Cancer of the Liver Italian Program (staging system); TNM: tumor-node-metastasis (staging system).

### Association of PCDH20 expression with patient survival

**Figure 3:**
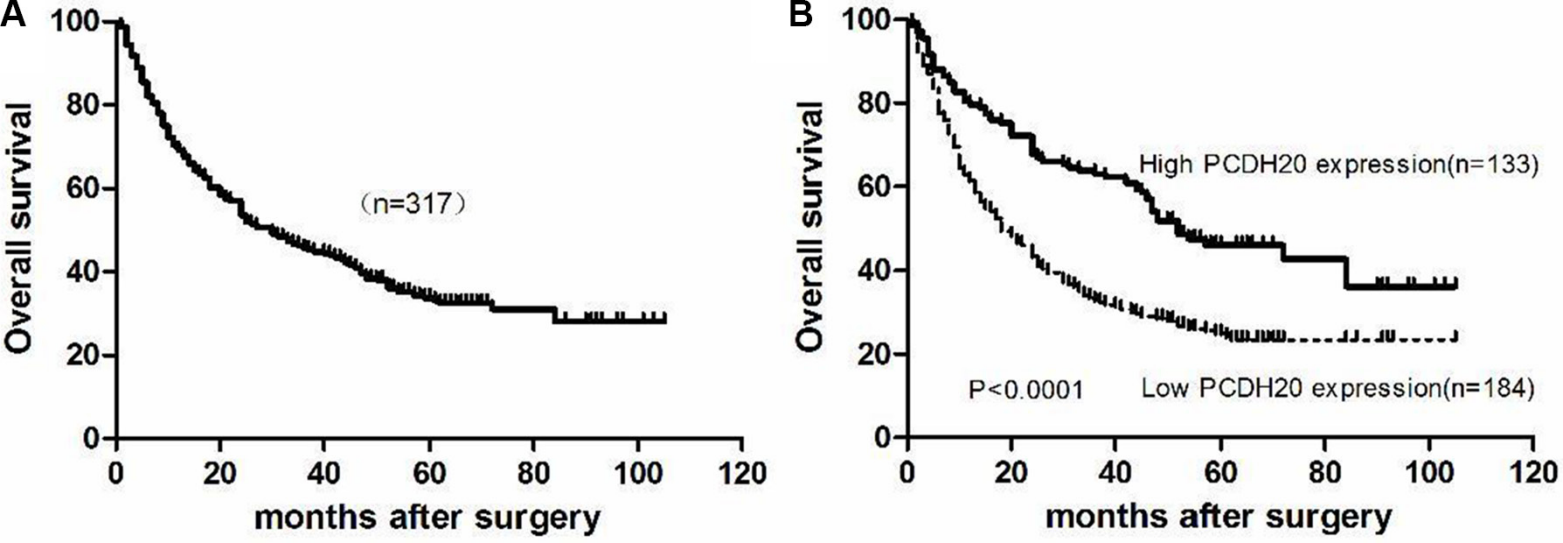
Kaplan-Meier curves for overall survival of hepatocellular carcinoma patients after surgical resection. (**A**) All HCC patients. (**B**) HCC patients with low vs. high PCDH20 expressions.

All patients were followed-up for a median of 30.0 months (Figure 3A), and the median follow-up periods in the high and low PCDH20 expression groups were 52.0 and 18.0 months, respectively. The 5-year OS rate in the low PCDH20 expression group was significantly lower than that in the high PCDH20 expression group (23.4% vs. 36.2%, respectively, *P* < 0.0001, Figure 3B).

**Figure 4:**
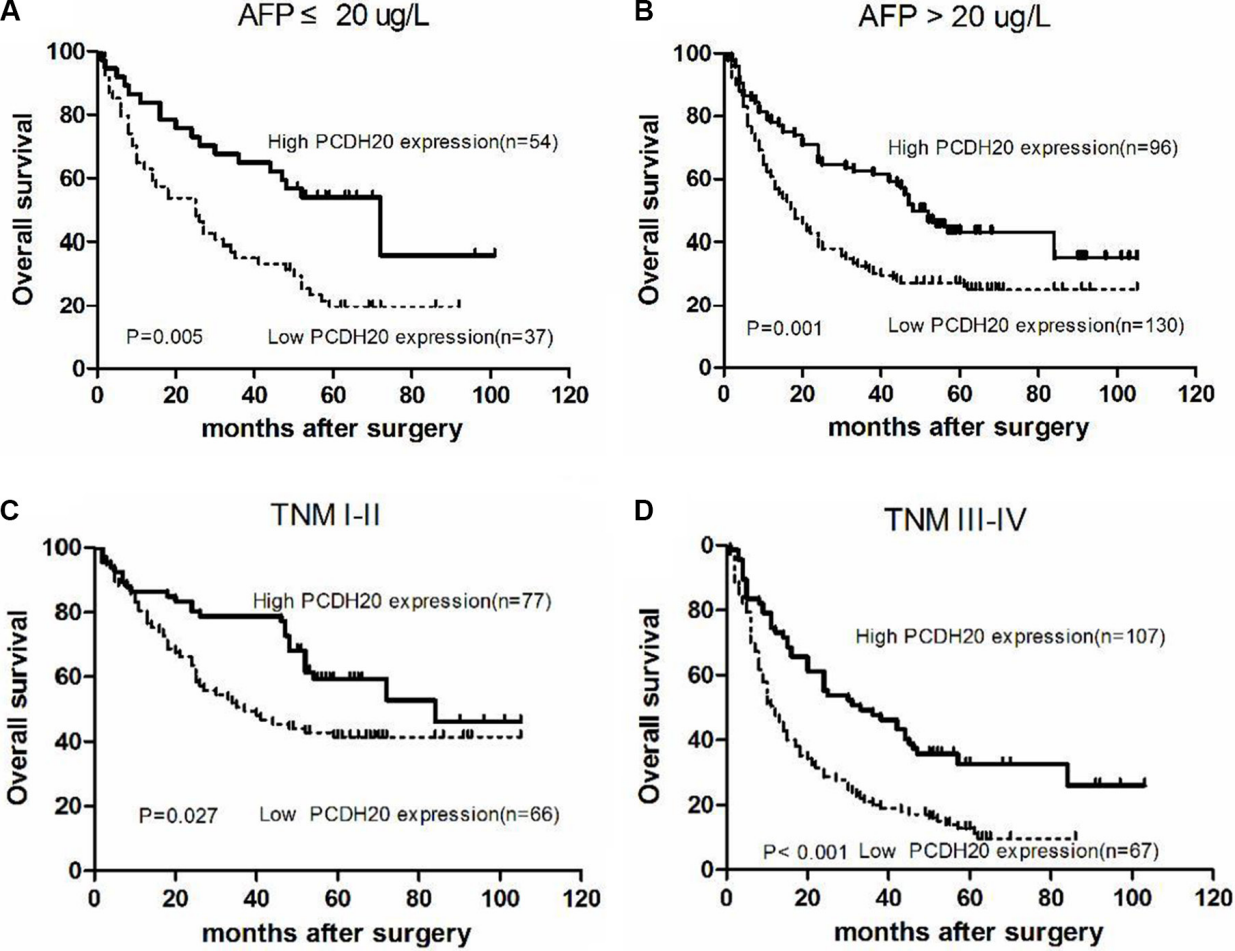
Kaplan-Meier curves for overall survival according to different AFP levels and TNM stages in hepatocellular carcinoma patients. (**A**) Alpha-fetoprotein (AFP) ≤ 20 μg/L, (**B**) AFP > 20 μg/L, (**C**) tumor-node-metastasis (TNM) stage I–II, and (**D**) TNM stage III-IV.

Additional sub-analyses were performed according to different alpha-fetoprotein (AFP) level and tumor-node-metastasis (TNM) stage. The results indicated that patients with low PCDH20 expression had worse OS than those with high PCDH20 expression in both patients with AFP ≤ 20 μg/L (*n* = 91) and AFP > 20 μg/L (*n* = 226) (Figure 4A, 4B). Similarly, in the subgroup analysis of different TNM stages, patients with high PCDH20 expression achieved more favorable OS than those with low PCDH20 expression for both patients with TNM I-II (*n* = 143) and TNM III-IV disease (*n* = 174) (Figure 4C, 4D).

### Univariate and multivariate analyses of prognostic variables in HCC patients

Univariate analysis showed that high aspartate aminotransferase (*P* = 0.005) and AFP (*P* = 0.020) levels and low PCDH20 expression (*P* = 0.020) were associated with poor OS (Table 3). In the multivariate analysis, low PCDH20 expression (*P* = 0.018), female sex (*P* = 0.010), multiple tumors (*P* = 0.043), and high AFP level (*P* = 0.048) emerged as independent risk factors for OS. To avoid the interference of collinearity factors, scoring systems such as the TNM and BCLC stage were excluded from the additional analyses, because such systems rely on tumor number, tumor size, and vascular invasion.

**Table 3:** Univariate and multivariate Cox regression analyses for overall survival in hepatocellular carcinoma

	Univariate analysis		Multivariate analysis		
	HR (95%CI)	*P* value	HR (95%CI)	*P* value	β
Sex (male vs. female)	1.409 (0.946~2.099)	0.091	1.992 (1.183~3.352)	0.010*	0.689
Age ( ≤ 50 vs. > 50 years)	1.004 (0.992~1.016)	0.501	1.095 (0.770~1.557)	0.614	0.090
Tumor differentiation (well vs. poor)	0.996 (0.709~1.399)	0.982	0.896 (0.590~1.362)	0.608	−0.110
Tumor number (solitary vs. multiple)	1.285 (0.966~1.710)	0.085	1.469 (1.013~2.132)	0.043*	0.385
Maximum tumor size ( ≤ 5 vs. > 5 cm)	1.501 (0.953~2.364)	0.080	1.446 (0.797~2.622)	0.225	0.369
Vascular invasion (absent vs. present)	0.928 (0.677~1.272)	0.642	0.922 (0.631~1.348)	0.675	−0.081
Portal hypertension (absent vs. present)	0.866 (0.483~1.551)	0.628	0.914 (0.481~1.736)	0.783	−0.090
Liver cirrhosis (absent/mild vs. moderate/severe)	1.198 (0.895~1.602)	0.224	0.829 (0.580~1.187)	0.307	−0.187
HBsAg (absent vs. present)	1.145 (0.797~1.64)	0.464	1.154 (0.728~1.829)	0.542	0.143
ALT ( ≤ 40 vs. > 40 U/L)	1.238 (0.945~1.622)	0.121	0.987 (0.668~1.457)	0.946	−0.014
AST ( ≤ 37 vs. > 37 U/L)	1.516 (1.135~2.024)	0.005*	1.335 (0.864~2.061)	0.193	0.289
TB (μmol/L)	1.000 (0.999~1.002)	0.622	1.001 (0.998~1.003)	0.621	0.001
ALB (g/dL)	1.011 (0.989~1.033)	0.341	1.010 (0.981~1.039)	0.504	0.010
INR ( ≤ 1.15 vs. > 1.15)	1.058 (0.788~1.421)	0.705	0.961 (0.656~1.409)	0.840	−0.039
CEA ( ≤ 5 vs. > 5 μg/L)	0.772 (0.475~1.097)	0.127	1.351 (0.861~2.120)	0.190	0.301
CA19-9 ( ≤ 35 vs. > 35 U/mL)	1.066 (0.760~1.496)	0.710	0.973 (0.660~1.433)	0.888	−0.028
CA125 ( ≤ 35 vs. > 35 U/mL)	1.226 (0.848~1.772)	0.279	1.235 (0.819~1.862)	0.313	0.211
AFP ( ≤ 20 vs. > 20 μg/L)	1.450 (1.059~1.985)	0.020*	1.450 (1.003~2.095)	0.048*	0.371
PCDH20 (high vs. low)	1.378 (1.052~1.805)	0.020*	1.542 (1.078~2.205)	0.018*	0.433

Abbreviations: HR: hazard ratio; CI: confidence ratio; HBsAg: hepatitis B surface antigen; ALT: alanine aminotransferase; AST: aspartate aminotransferase; TB: total bilirubin; ALB: albumin; INR: international normalized ratio; CEA: carcinoembryonic antigen; CA19-9: carbohydrate antigen 19–9; CA125: carbohydrate antigen 125; AFP: alpha-fetoprotein; PCDH20: protocadherin 20.

### A simple risk score for predicting HCC patient survival

**Figure 5:**
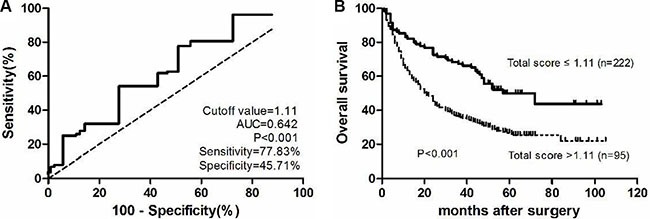
A novel simplified risk score. (**A**) Receiver operating characteristics curve for the simplified risk score. (**B**) Kaplan-Meier curves for overall survival of different simplified risk scores.

In our cohort, sex, PCDH20, AFP, and tumor number were found to be four crucial independent prognostic factors for HCC. To identify a better significant prognostic model, we developed a risk score from the weighted sum of these four variables in the multivariate Cox regression model. A clinicopathological prognostic nomogram was generated as follows: score = 1.86 × (1 if female) + 1.04 × (1 if multiple tumors) + 1.17 × (1 if low PCDH20 expression) + 1.00 × (1 if AFP > 20 μg/L). The total score ranged from 0 to 5.07. The optimal cut-off value was determined as 1.11 by receiver operating characteristic curve analysis and the highest Youden index value (Figure 5A). Using this cut-off value, the sensitivity and specificity of death prediction in HCC patients after surgery were 77.83% and 45.71%, respectively. The area under the receiver operating characteristic curve was 0.642 (Figure 5A). Patients with a total score > 1.11 had a poorer OS than those with scores ≤ 1.11 (*P* < 0.001, Figure 5B). Finally, by comparing Harrell's concordance index (C-index), we found that our simple risk score had higher diagnostic accuracy for predicting HCC survival than the other scoring systems depicted in Table 4, except for the CLIP score (Table 4).

**Table 4:** Ranking of predictive abilities of the prognostic systems according to the C-index

Rank	System	C-index	95% CI
1	CLIP score	0.635	0.593–0.677
2	Risk score based on PCDH20	0.614	0.572–0.656
3	BCLC stage	0.591	0.549–0.633
4	Okuda stage	0.573	0.531–0.615
5	Child-Pugh classification	0.533	0.491–0.575

Abbreviations: CI: confidence interval; CLIP: Cancer of the Liver Italian Program (staging system); PCDH20: protocadherin 20; BCLC: Barcelona Clinic Liver Cancer (staging system).

## DISCUSSION

In the current study, the mRNA and protein expressions of PCDH20 were significantly reduced in HCC tissues and cell lines compared to that in PC tissues and normal human hepatocyte, as determined by quantitative real-time polymerase chain reaction and western blot. Furthermore, PCDH20 protein expression in HCC tissues was significantly lower than in the adjacent normal tissues, as confirmed by immunohistochemistry of a large independent cohort of clinical specimens. Decreased PCDH20 expression correlated with portal hypertension, poor tumor differentiation, advanced Okuda stage, and CLIP score, with patients with lower PCDH20 expression having a higher risk of mortality.

AFP is a well-established biomarker for the diagnosis and monitoring of HCC; however, its practical value has been questioned due to its poor sensitivity and specificity [22, 23]. Further, only 60% of HCCs produce AFP [24]. Identification of aggressive tumors from indolent ones is critical for optimization of individualized treatments. Thus, in this study, we assessed whether PCDH20 could predict prognosis in different subgroups based on AFP. As expected, patients with high PCDH20 expression showed more favorable OS than those with low PCDH20 expression in both the normal and abnormal AFP subgroups. Meanwhile, individuals with the same tumor stage often present various clinical outcomes, owing to the heterogeneity of the genetic alterations present. In early-stage HCC, it is usually hard to predict outcomes by conventional indicators. However, in the subgroup analysis by TNM stage, the predictive value of PCDH20 was similar irrespective of whether early- or late-stage tumors were evaluated. Taken together, these results indicate that PCDH20 is a sensitive clinical parameter for predicting survival of indolent and early-stage cases.

Based on the finding that PCDH20 was a favorable maker for HCC prognosis, we next combined it with other three significant clinical variables to develop a simple risk score, and found that this risk score showed better predictive ability than BCLC stage, Okuda stage, and Child-Pugh classification. For patients with a risk score > 1.11, more intense follow-up and adjuvant therapy administration may be warranted after initial surgery, and personalized therapeutic regimens should also be considered. In fact, we recently observed that overexpression of PCDH20 relates to chemosensitivity to cisplatin in HCC cells (unpublished data), suggesting that PCDH20 may represent a potential, useful therapeutic target of HCC.

In a recent study, reduced PCDH20 mRNA expression was found to only be associated with younger age in HCC patients [20], which is inconsistent with our findings. The reason for this discrepancy may be that the previous study measured only the mRNA levels, whereas the PCDH20 protein expression was additionally investigated in our study. Moreover, many common tumor biomarkers (such as carbohydrate antigen (CA) 125, CA19-9, and carcinoembryonic antigen) have been reported to be predictive of prognosis in HCC accompanied by portal vein tumor thrombosis [25], but none of these markers was found to show a predictive role or to be related to the expression of PCDH20 among the HCC patients in our study. Possible explanations might be that the serum carcinoembryonic antigen, CA19-9, and CA125 levels were normal in most cases in the current study, which was similar to the previous study [26]. Besides, this was a single-center retrospective study, which might have resulted in selection bias.

There is a paucity of data regarding the underlying mechanism of PCDH20 activity in the cancer setting. It has been reported that the frequent silencing of PCDH20 in non-small cell lung carcinoma cell lines and primary tumors was associated with promoter methylation, and that tumor cell growth was suppressed after restoration of PCDH20 expression *in vitro* [18]. In nasopharyngeal carcinoma, PCDH20 was identified as a functional tumor suppressor via inactivation of Wnt/β-catenin signaling and epithelial-to-mesenchymal transition, with frequent epigenetic inactivation observed [19], while another study on HCC revealed that hypermethylation of the PCDH20 promoter accounted for its downregulation; moreover, overexpression of PCDH20 could inhibit cell proliferation and cell migration by antagonizing the Wnt/β-catenin signaling pathway [20]. Nevertheless, detailed understanding of the functions and mechanisms of PCDH20 remains limited, warranting further studies.

There are several limitations in this study. First, the data were obtained from a single center, and the sample size was limited. Second, selection bias existed in the study; for example, we included only patients with resectable tumors. Early-stage patients treated with interventional therapy or advanced cases subjected to palliative treatment were excluded. This might explain why some important pathological features, such as the tumor pathological grade and maximum tumor size, were not independent risk factors for HCC prognosis in our study. A multicenter study incorporating a larger number of patients and with a prolonged observation time is required to confirm our findings.

In conclusion, low expression of PCDH20 was found to be associated with poor OS in HCC patients; hence, this protein represents a promising potential prognostic biomarker. Moreover, our novel risk score based on PCDH20 appears to represent a reliable predictor of HCC patient survival, and may therefore be useful for providing guidance for clinical management.

## MATERIALS AND METHODS

### Patients, tissue samples, and follow-ups

A total of 317 HCC patients who underwent partial hepatectomy at the First Affiliated Hospital, Sun Yat-sen University, between January 2004 and December 2009 were enrolled in the study. Patients who received other treatments (transarterial chemoembolization, chemotherapy, or radiofrequency ablation) before surgery, or had other malignant diseases were excluded. PC tissues were defined as tissues located 2–5 cm from the tumor border [27]. Tumor differentiation was based on the Edmondson classification [28]. Tumor stage was determined according to the 6th edition of the TNM classification of the American Joint Committee on Cancer [29]. Ethical approval for this study was granted by the Ethics Committee of the First Affiliated Hospital, Sun Yat-sen University.

Patient follow-up data were obtained after discharge by contacting the patients or other relatives via telephone, or by reviewing their hospital records. All patients were followed-up until death or until censoring on December 1, 2013.

### Quantitative real-time polymerase chain reaction

The immortalized human fetal liver cell line LO_2_ and the human liver cancer cell lines Huh-7, HepG2, SMMC7721, and SK-hep-1 were maintained in Dulbecco's Modified Eagle Medium supplemented with 10% fetal bovine serum (Gibco, Grand Island, NY, USA). All cell lines were maintained in a humidified incubator containing 5% CO_2_ at 37°C. All fresh tumors and matched PC specimens were immediately stored on dry ice after resection and were subsequently frozen at −80°C. Total RNA was extracted from clinical samples or cell lines using Trizol reagent (Invitrogen, Carlsbad, California, USA). cDNA was synthesized using a first strand cDNA synthesis kit (Roche, Penzberg, Germany), as previously described [30]. The mRNA levels were analyzed using Fast Start Universal SYBR Green Master (Roche). Three replicates were taken for each sample. GAPDH mRNA was used as the internal control for PCDH20. The primers for PCDH20 were 5′-AAGGGTATGCTGAGGGCTAAA-3′ (forward) and 5′-GGAAACAAAACAAGAGGAGGGT-3′ (reverse). The primers for GAPDH were 5′-CGCTGAGTACGTCGTGGAGTC-3′ (forward) and 5′-GCTGATGATCTTGAGGCTGTTGTC-3′ (reverse).

### Western blot analysis

Total proteins were extracted from cells and tissues using lysis buffer, as described previously [31]. Protein lysates were separated on 10% sodium dodecyl sulfate polyacrylamide gels and subsequently transferred onto polyvinylidene fluoride membranes. After blocking with 5% skim milk in Tris-Buffered Saline-Tween 20 for 2 hours, membranes were incubated with PCDH20 antibody (1:500; Abcam, Cambridge, UK) or GAPDH antibody (1:1000; Cell Signaling Technology, Danvers, MA, USA) at 4°C overnight. Next, the membranes were washed in Tris-Buffered Saline-Tween 20 and exposed to anti-rabbit immunoglobulin G horseradish peroxidase-conjugated antibody (1:3000, Cell Signaling Technology) for 1 hour at room temperature. Band signals were visualized using the Image Quant Las 4000 Mini system (GE Healthcare, Stockholm, Sweden). All experiments were performed in at least triplicates.

### Immunohistochemistry staining

Formalin-fixed, paraffin-embedded tissue sections were used for immunohistochemistry. After antigen retrieval, the endogenous peroxidase activity was blocked in hydrogen peroxide (0.3%). Next, the slides were incubated with anti-PCDH20 antibody (1:200; Santa Cruz) overnight at 4°C, followed by incubation with biotinylated goat anti-rabbit/mouse antibodies at room temperature after rinsing in phosphate-buffered saline (pH 7.2). Negative control slides were incubated in parallel in phosphate-buffered saline only. Finally, the slides were counterstained with hematoxylin, dehydrated, and mounted in resin blocks. Five high-power fields were randomly chosen for assessment of PCDH20, and at least 300 cells were counted per field. Two independent pathologists evaluated the immunostaining. Each tumor section was assigned a score according to the intensity of the staining and the proportion of stained tumor cells. The intensity of staining was scored as 0 (negative), 1 (weak), 2 (moderate), or 3 (strong). The extent of staining was scored based on the percentage of positive tumor cells: 0 (0%), 1 (0–10%), 2 (10–50%), and 3 (50–100%). The two scores were multiplied, resulting in final scores ranging from 0 to 9 [32]. For statistical analysis, scores of 0–4 were considered low expression, while scores of 5–9 were considered high expression; the cut-off was determined by receiver operating characteristics curve analysis.

### Statistical analysis

Student's *t*-test was used to evaluate differences in the protein and mRNA expressions between the HCC and PC tissues. The relationship between PCDH20 expression and clinicopathological parameters were assessed by chi-square test. The prognostic value of PCDH20 expression on patient survival was calculated by the Kaplan-Meier method and log-rank tests. Univariate and multivariate Cox proportional hazard models were used to determine the independent prognostic factors for HCC. A risk score was designed by using the significant variables derived from the multivariate Cox regression analysis (i.e., factors with *P* < 0.05). The prognostic value of the HCC prognosis prediction was determined by comparing Harrell's C-index. For all analyses, *P* values < 0.05 were considered statistically significant. The statistical analyses were performed using SPSS version 19.0 (SPSS Inc., Chicago, IL, United States) and GraphPad Prism version 5.0 software.
